# Referrals for rheumatologic evaluation following a positive antinuclear antibody test result. A cross-sectional single center Brazilian study

**DOI:** 10.1186/s42358-024-00390-y

**Published:** 2024-06-30

**Authors:** Leonardo Borgato Della Vecchia, Caio Delano Campos Oliveira Assis, Fernando de Oliveira Salatiel, Maria Thereza Santos Cirino, Maria Eduarda Vogel Scarpante, Vanessa Monteiro Oliveira, Letícia Pedroso Meneghin, Maria Júlia Gonçalves Silva, Victória Ferini dos Santos, Natália Pavoni Catardo, Isabela Pulini Nemesio, Lívia Loamí Ruyz Jorge de Paula, Carolina Borges Garcia Sasdelli, Ana Beatriz Santos Bacchiega

**Affiliations:** 1 Faculdade de Ciências da Saúde de Barretos Dr Paulo Prata, Av. Loja Maçonica Renovadora N° 68, n° 100, Barretos - SP, CEP: 14785-002 Brazil; 2 Ambulatório Médico de Especialidades de Barretos (AME), Av. Loja Maçonica Renovadora 68, n° 105, Barretos - SP, CEP: 14785-002 Brazil

**Keywords:** Rheumatology, Referral, Systemic lupus erythematosus, Antinuclear antibody

## Abstract

**Background:**

In general, patients are referred for rheumatological evaluation due to isolated laboratory abnormalities, especially antinuclear antibody (ANA) positivity, with the risk of more severe patients remaining on the waiting list for longer than desired. The aim of this study was to analyze the demographic, clinical, and laboratory information of patients referred to a specialized rheumatological care unit because of positive antinuclear antibody.

**Methods:**

This is a retrospective study of 99 out of 1670 patients seen by the same rheumatologist between 01/01/2011 and 01/01/2019. Patients whose referrals were exclusively due to the ANA test result and the specialist’s final diagnosis being “abnormal finding of serum immunological test” (ICD-10 R769) were included. Sociodemographic, clinical, and laboratory information were extracted from the consulting rheumatologist’s chart. Descriptive statistics were used for data analysis.

**Results:**

A total of 99 patients were included, most of whom were female (84.8%) with a median age of 49 years. At the moment of specialist’s appointment, 97 patients (97.9%) repeated the ANA test, and 77 patients remained positive. Of these, only 35 (35.35%) were in a high titer range (greater than or equal to 1:320). Complete blood count for cytopenia’s investigation was not performed in a high percentage of patients (22.2%), as well as urinalysis (31.3%). In addition, more than 70% of patients score 0 to 1 classification criteria for Systemic Lupus Erythematosus, according to SLE - ACR 1987 (American College of Rheumatology) and SLICC 2012 (Systemic Lupus International Collaborating Clinics).

**Conclusions:**

Most patients are still referred for specialized evaluation due to the misinterpretation of laboratory tests that were inappropriately requested in patients without clinical evidence of autoimmune rheumatic disease.

## Background

The waiting time for rheumatology care in the Brazilian Public Health System is variable and could be long such as almost a year [1]. As a result, it can lead to a delay in the diagnosis of systemic inflammatory rheumatological diseases with consequent social and economic impacts.

There is a concern regarding the better management of referrals to the specialist, considering that most of cases of fibromyalgia, osteoarthritis, and gout are referred for a rheumatological evaluation but could be followed in a primary health care.

Strategies are needed to improve the waiting list and therefore protocols with telemedicine can play a role in reducing the inappropriate referrals to the specialist [2]. About half of patients referred had conditions that do not meet autoimmune rheumatic diseases criteria [2]. It is not well established what are the possible causes for this situation, but it seems to be most likely a multifactorial problem, including the lack of recognition of prevalent diseases and inadequate workup.

Isolated laboratory abnormalities, like a positive ANA, are referred for rheumatological evaluation as a result of inadequate requests for complementary tests in primary health care services. Moreover, the use of HEp-2 cells as a substrate for performing ANA increased sensitivity, resulting in more false positive or clinically irrelevant results [3]. Thus, this study aims to recognize the demographic, clinical, and laboratory aspects of patients referred to a rheumatological care service because of positive antinuclear antibody.

## Methods

This is a retrospective study of 99 out of 1670 outpatients, men and women aged 18 years or older, referred to Ambulatório Médico de Especialidades de Barretos (AME) for rheumatological evaluation over an eight-year period (2011–2019) and assessed by the same specialist. Patients that presented with abnormal ANA results and who received the diagnosis of “abnormal finding of serum immunological examination” (ICD-10 R769) after specialized medical evaluation were selected. Patients eventually diagnosed with an autoimmune disease were excluded from the study.

The study was approved by the research ethics committee of the Pio XII Foundation, and all participants agreed to participate by giving consent by telephone, following the guidelines of Brazilian ethical resolutions, as well as the Declaration of Helsinki. The collected data were entered into the REDCap platform (Research Electronic Data Capture), and statistical analyses were performed using SPSS software version 27.0 [4].

Data were collected from the electronic medical form to record demographic information (gender and age) and clinical information (smoking, serology for hepatitis B, hepatitis C or HIV, rheumatoid factor, and ASLO - anti-streptolysin O). Most of the patients repeated ANA at our central laboratory and a dilution equal to or greater than 1:320 was considered a high titer [5].

Clinical and laboratory data were applied to the classification criteria for systemic lupus erythematosus published by the American College of Rheumatology (ACR) and Systemic Lupus International Collaborating Clinics (SLICC): arthritis/arthralgia, photosensitivity, serositis, alopecia, malar rash, oral ulcers, seizure/psychosis or other neuropsychiatric changes, skin changes, cytopenias, and glomerulonephritis [6–8].

The patients were also scored to SLE Risk Probability Index **(**SLERPI) to access SLE risk probability. This is an algorithm based on classical disease features that was developed based on machine learning and validated. This tool was proposed for general practitioners and it is useful in accessing SLE risk probability (unlikely, could not be rule out, likely and definite).

Regarding autoimmunity related laboratory data, the results of anti-dsDNA, anti-Sm, complement (C3, C4, Ch50), antiphospholipid (lupus anticoagulant, anticardiolipin IgM and IgG) were reviewed [6–8], as well as the presence of anti-Ro (anti-SSA) and anti-La (anti-SSB) antibodies.

Descriptive statistics were used for data analysis, such as measures of central tendency (mean and median), dispersion measures (standard deviation, maximum and minimum), and absolute and relative frequencies.

## Results

We included 99 patients who were referred due to changes in the ANA test requested by referring physician and who, after being evaluated by a rheumatologist, were discharged. Of these patients, 84 patients (84.8%) were female with a median age of 49 years (ranged between 20 and 93 years) and among them,17 were 65 years old or older. Almost 80% of patients were nonsmokers (76.9%).

Most of the patients were not evaluated regarding chronic infections, as shown in Table 1. Only two patients (2%) had positive serology for hepatitis C virus, and one patient (1%) had HIV chronic infection.

**Table 1:** Results of serologies performed

Serology	Negative	Positive	Not evaluated
HBsAg	36 (36.4%)	–	63 (63.6%)
Anti– Hbs	7 (7.1%)	–	92 (92.9%)
Anti– Hbc	7 (7.1%)	–	92 (92.9%)
Anti– HCV	40 (40.4%)	2 (2%)	57 (57.6%)
Anti– HIV	14 (14.1%)	1 (1%)	84 (84.9%)

*HBsAg* hepatitis B virus surface antigen, *Anti Hbs* antibody against the surface antigen of the hepatitis B virus, *Anti Hbc* antibody against the core antigen of the hepatitis B virus, *Anti HCV* antibody against the hepatitis C virus, *anti HIV* antibody against the human immunodeficiency virus

Seventeen patients (17.2%) were referred with the ASLO results already requested at the origin; of these, only 4 had positive results (4%). Rheumatoid factor was performed in 40 patients (40.4%), with most of these results being negative (92.5%).

In the evaluation with the specialist, 97 patients (97.9%) repeated the ANA, and 77 patients remained positive. Of these, only 35 patients (35.35%) had a high ANA titer (greater than or equal to 1/320). The distribution of HEp-2 immunofluorescent assay (IFA) ANA patterns is shown in Table 2.

**Table 2:** Distribution of HEp-2 (IFA) ANA patterns

ANA pattern	*N* = 77
Dense fine speckled nuclear	32 (41.5%)
Fine speckled nuclear	13 (16.8%)
Homogeneous nuclear	21 (27.2%)
Coarse speckled nuclear	3 (3,8%)
Homogeneous nucleolar	1 (1,2%)
Cytoplasmic	2 (2.5%)
Centromeric	1 (1.2%)
Mixed fine speckled and homogeneous nucleolar	2 (2.5%)
Mixed coarse speckled and reticular cytoplasmic	1 (1.2%)
Pattern not described in medical records	1 (1.2%)

*HEp-2 IFA* immunofluorescent assay on HEp2 cells, *ANA* antinuclear antibody

There was no protocol for workup of patients referred from primary care and the exams were requested at the discretion of the attendant rheumatologist. After rheumatological evaluation, a percentage of patients did not even undergo a complete blood count to investigate cytopenia (22.2%) and urinalysis to assess urinary sediment (31.3%).

Blood count analysis is fundamental for the evaluation of the hematological activity of SLE and regarding this clinical manifestations, nine patients (9.1%) had cytopenia. However, no patient had hemolysis. Among them, three (33%) had fatty liver disease (alcoholic and nonalcoholic) and one (11%) patient had primary biliary cholangitis, all of them with portal hypertension. One (11%) was diagnosed with Sjögren’s syndrome, one (11%) was diagnosed with visceral leishmaniasis, one (11%) had a history of bariatric surgery associated with previously documented B12 hypovitaminosis, one (11%) had co infection HIV and HCV (Table 3). Portal hypertension could justify the hematological findings in 4 out of 9 patients. Although ordering ANA is part of the investigation of patients with liver cirrhosis to rule out autoimmune hepatitis [9], this test was requested to assess cytopenia and not to investigate chronic liver disease, and patients were diagnosed with portal hypertension only in rheumatological appointment [10].

Among the two patients who had hematuria in routine urinalysis, one patient had a urinary tract infection (with a positive urine culture), and the other patient had a history of nephrolithiasis. None of the cases presented with dysmorphic hematuria.

**Table 3:** Laboratorial findings of cytopenic patients

ANA (AME)	ANA pattern	Cytopenia	Autoantibodies	Comorbidities
Negative		Leukopenia/thrombocytopenia	Acl IgM/IgG negative, LA negative, anti-beta2GPI IgM/IgG negative	Fatty liver disease
1/320	Homogeneous nuclear	Leucopenia	Anti Ro negative, anti La negative, anti Sm negative, anti DNA negative	
Negative		Leukopenia/lymphocytopenia/thrombocytopenia	Acl IgM/IgG negative, LA negative	Cirrosis
1/320	Mixed coarse speckled and reticular cytoplasmic	Leukopenia/lymphocytopenia/thrombocytopenia	Acl IgM/IgG negative, anti-beta2GPI IgM/IgG negative, LA negative, anti Ro negative, anti La negative, anti Sm negative, anti DNA negative	primary biliary cholangitis/HCV infection
1/80	Homogeneous nuclear	Leukopenia/thrombocytopenia	NA	AIDS/HCV infection
1/80	Dense fine speckled nuclear	Leucopenia	Acl IgM/IgG negative, anti-beta2GPI IgM/IgG negative, LA negative, anti Ro negative, anti La negative, anti Sm negative, anti DNA negative	
1/80	Coarse speckled nuclear	Leukopenia/thrombocytopenia	Acl IgM/IgG negative, anti-beta2GPI IgM/IgG negative, LA negative, **anti Ro 240**, anti La negative, anti Sm negative, anti DNA negative	Visceral leishmaniasis
1/160	Fine speckled nuclear	Leucopenia	Acl IgM/IgG negative, anti-beta2GPI IgM/IgG negative, LA negative, **anti Ro 240**, anti La negative, anti Sm negative, anti DNA negative	Bariatric surgery/hypovitaminosis B12
1/160	Fine speckled nuclear	Leukopenia/thrombocytopenia	Acl IgM/IgG negative, anti-beta2GPI IgM/IgG negative, LA negative, anti Ro negative, anti La negative, anti Sm negative, anti DNA negative	Cirrosis

*Acl IgM/IgG* anticardiolipin antibodies, *anti-beta2GPI IgM/IgG* anti-beta2 glycoprotein I antibodies, *HCV infection* chronic hepatitits C virus infection, *AIDS* acquired immunodeficiency syndrome, *HBsAg* hepatitis B virus surface antigen, *Anti Hbs* antibody against the surface antigen of the hepatitis B virus, *Anti Hbc* antibody against the core antigen of the hepatitis B virus, *Anti HCV* antibody against the hepatitis C virus, *anti HIV* antibody against the human immunodeficiency virus, Bold values mean abnormal results

Although some patients were tested for anti-DNA (66.6%) and anti-SM (60.6%) requested by the rheumatologist, none of them were positive for these antibodies. However, 94 patients (94.9%) did not undergo investigation of the C3 and C4 complement cascade fractions after rheumatological evaluation, and consumption was not identified in those patients who were tested. Most patients (96%) were not tested to antiphospholipid antibody testing. The presence of anti-Ro (anti SSA) and anti-La (anti SSB) antibodies was tested in 64 patients (64.64%), with only 4% positivity for the anti Ro.

Most patients had only ANA positivity as a classification criterion for systemic lupus erythematosus. Other criteria presented by the patients in the study were distributed as follows: seven patients (7%) had photosensitivity, six patients (6%) had nonscarring alopecia, three patients (3%) had malar rash, two patients (2%) had polyarthralgia, one patient (1%) had kidney damage, one patient (1%) had oral ulcer and one patient (1%) had central nervous system disorders (convulsive crisis). Tables 4 and 5 present data on the distribution of patients according to the fulfillment of the classification criteria of SLE. Few patients in this study were tested for chronic viral infections. Two patients met the 4 classification criteria for SLE but had other clinical conditions that justified the manifestations presented. The patient with the maximum score in the 1987 ACR criteria also had photosensitivity, known as a manifestation with lower specificity for SLE. Those who scored the 2012 SLICC criteria were diagnosed as coinfection HIV and hepatitis C only at the rheumatologist’s appointment, which justified the laboratory alterations presented (leukocyturia, hematuria, leukopenia, and thrombocytopenia), as well as the complaint of oral ulcers.

**Table 4:** Distribution of patients according to fulfilment of the classification criteria of the SLE - ACR 1987 (*American College of Rheumatology)*

The number of criteria scored	*N*	%	Confidence interval
0 ou 1	79	79.8	(0.719, 0.877)
2	16	16.2	(0.089, 0.235)
3	3	3	(−0.004, 0.064)
4	1	1	(− 0.01, 0.03)
Total	99	100	

**Table 5:** Distribution of patients according to the fulfilment of the classification criteria of the SLICC 2012 (*Systemic Lupus International Collaborating Clinics)*

The number of criteria scored	*N*	%	Confidence interval
0 ou 1	71	71.7	(0.628, 0.806)
2	22	22.2	(0.14, 0.304)
3	5	5.1	(0.008, 0.094)
4	1	1	(0.01, 0.03)
Total	99	100	

After applying SLERPI, 91 patients (92%) were scored as unlikely SLE. Lupus could not be rule out in one patient (1%), three patients (3%) scored as likely and four (4%) patients scored as definite SLE.

## Discussion

In the present study, we identified that approximately 6% of the patients referred to rheumatologist assessment were due to an isolated ANA result. Most of these patients did not present any other classification criteria (clinical or laboratory) for systemic lupus erythematosus (SLE). This data is similar to that found in another study [11]. The unproperly requesting of exams and their misinterpretation in primary care could impact in a high number of unnecessary referrals, that delay the access of patients with severe diseases to the specialist.

A large percentage of patients who underwent the ANA testing did not undergo easily accessible tests that are essential in suspected SLE, such as blood count and urine1 to assess hematological and renal manifestations, respectively. Considering this diagnostic hypothesis, it is mandatory to rule out severe manifestation and/or document them (hemolysis and glomerulonephritis) to refer promptly to hospital admission, for example.

In addition, complementary request for autoantibodies in the investigation of autoimmunity requires correlating them with epidemiology and symptoms presented. Late-onset SLE is described in patients older than fifty years-old but the referral of a 93-year-old patient with suspected of autoimmunity is noteworthy, considering that this is not the age range usually affected by the disease [12]. Ordering ASLO, rheumatoid factor, and ANA for the same patient reinforces the fact that there is no clear diagnostic hypothesis for the presented complaints and the patients were evaluated with “rheumatological tests” as a whole [13].

Although the ANA is not used to monitor disease activity, patients repeated the test at the AME laboratory due to the lack of reliability of the result. It is clear that more than 70% of patients did not have any other classification criteria (ACR 1987 and SLICC 2012) for SLE other than ANA positivity. That is, autoantibodies were requested for the diagnostic investigation even in the absence of clinical findings that would require them. Before applying such criteria, careful clinical judgment is needed, and exclusion of autoimmunity mimics including infectious, metabolic, and neoplastic diseases is necessary [8, 14].

It is noteworthy that non rheumatologists may not be familiar in diagnosing SLE, and that is the reason why SLERPI was developed [15]. According to this tool, four patients in this study would be considered as definite SLE, but their hematological score were due to portal hypertension. If this approach was used at primary care, more than 90% of these patients initially diagnosed as being “abnormal finding of serum immunological test” would not be referred to specialized appointment.

Another relevant aspect is the distribution of the ANA title and patterns. In this study, there was a higher prevalence of low ANA titers (1/80 and 1/160), which are often associated with healthy individuals [16]. Interestingly, both patients with higher scores on the SLE classification criteria did not have a high ANA titer. In this study, the most frequent pattern was the dense fine speckled nuclear. Studies indicate that antibodies related to this pattern are not associated with the development of systemic rheumatological diseases and have a low prevalence in patients with SLE (1.1%) [16].

Our study has some limitations. One of them is the retrospective analysis of the medical records from one single-center. Most of the patients were already asymptomatic at the moment of the clinical assessment, making the identification of exact reason for referring physician requesting the ANA test difficult. Furthermore, there was no protocol for the exams required in the evaluation of those patients. They were performed depending on the judiciousness of the attendant rheumatologist and the workup of the other connective tissue diseases frequently presenting a positive ANA test were unavailable. Another limitation is that was not possible to review medical chart from all patients diagnosed with rheumatic disease attended at AME. Moreover, it is now well established that ANA and some disease-specific autoantibodies (anti-dsDNA antibodies in SLE, for example) can antedate the clinical diagnosis of autoimmune rheumatic disease by as many as two decades. Different HEp-2 IFA patterns indicate different autoantibodies and we must take it in count to judge its clinical relevance before advising patients that only some are associated with a specific disease [17, 18].

The strong point of our study is that it is a large national cross-sectional study of Brazilian Public Health System patients, which brought to light the weaknesses in the interpretation and propedeutics related to musculoskeletal complaints and the use of laboratory tests, resulting in inappropriate referrals and a waste of health care resources. Another point is that all patients were evaluated by the same experienced rheumatologist.

## Conclusion

An autoimmunity work-up is frequently requested for patients with musculoskeletal pain due to failure to recognize and manage soft tissue rheumatism, conditions that are much more prevalent in primary care. The pre-test probability of systemic autoimmune rheumatic disease must be taken to account before perform autoimmunity tests.

Thus, it appears that offering continuing medical education on the epidemiological data of diseases in different populations for the proper use of resources, as well as the interpretation of laboratory tests related to autoimmunity in health services, especially in primary care, would contribute to improving waiting time for a rheumatological visit. Furthermore, this study highlights the importance of better training for physicians, whether in primary or secondary care, when dealing with rheumatological conditions.

## Data Availability

The datasets used and/or analyzed during the current study are available from the corresponding author upon reasonable request.

## Abbreviations

ACR: American College of Rheumatology
AME: Ambulatório Médico de Especialidades
ANA: Antinuclear factor
ASLO: Anti-streptolysin O
ICD-10: International Statistical Classification of Diseases and Related Health Problems 10th Revision
HBsAg: Hepatitis B virus surface antigen
Anti-Hbs: Antibody against the surface antigen of the hepatitis B virus
Anti-Hbc: Antibody against the core antigen of the hepatitis B virus
Anti-HCV: Antibody against the hepatitis C virus
Anti-HIV: Antibody against the human immunodeficiency virus
SLE: Systemic lupus erythematosus
SLICC: Systemic Lupus International Collaborating Clinics
